# Redefining Systemic Sclerosis Classification: Anti-Topoisomerase Antibody as a Superior Predictor of Interstitial Lung Disease and Skin Progression Compared to Limited Cutaneous Systemic Sclerosis Subset

**DOI:** 10.3390/life15071067

**Published:** 2025-07-04

**Authors:** Chana Chaovanitkul, Tippawan Onchan, Patnarin Pongkulkiat, Ajanee Mahakkanukrauh, Siraphop Suwannaroj, Chingching Foocharoen

**Affiliations:** Department of Medicine, Faculty of Medicine, Khon Kaen University, Khon Kaen 40002, Thailand; ellechana@gmail.com (C.C.); tippon@kku.ac.th (T.O.); patnpo@kku.ac.th (P.P.); ajamah@kku.ac.th (A.M.); siraphop@kku.ac.th (S.S.)

**Keywords:** systemic sclerosis, scleroderma and related disorders, antigens and autoantibodies, observational studies, skin, respiratory, renal, cardiovascular

## Abstract

**Background:** Currently, no information exists on the clinical course of anti-topoisomerase I antibody (ATA)-positive limited cutaneous systemic sclerosis (lcSSc). We aimed to evaluate the incidence of and time to the development of interstitial lung disease (ILD), pulmonary hypertension (PHT), scleroderma renal crisis (SRC), and maximal modified Rodnan skin score (max-mRSS) in patients with lcSSc and dcSSc, with and without ATA. **Methods:** This cohort study included 522 patients with systemic sclerosis (SSc). The incidence of and time to the development of ILD, PHT, SRC, and max-mRSS were assessed. **Results:** ATA-positive dcSSc (dcSSc-posATA) was the most common presentation among Thai patients (321 cases; 61.5%). The median time to the development of ILD was shorter than that in lcSSc-posATA, comparable to that in dcSSc-posATA (1.0 vs. 1.8 years, *p* = 0.21), and shorter than that in ATA-negative dcSSc (dcSSc-negATA) (1.0 vs. 4.8 years, *p* = 0.001). The time to max-mRSS in lcSSc-posATA was comparable to that in dcSSc-posATA (*p* = 0.17) but shorter than that in dcSSc-negATA (*p* < 0.001). **Conclusions:** Patients with lcSSc-posATA had a similar risk of ILD development and time to reach max-mRSS as those with dcSSc, regardless of the presence of ATA, but had earlier ILD development and max-mRSS compared to those with dcSSc-negATA. Their prognosis appeared to be better than that of dcSSc-posATA.

## 1. Introduction

Systemic sclerosis (SSc) is an autoimmune disease associated with vasculopathy and fibrosis of internal organs. Patients with SSc can present with minor nonspecific manifestations, such as Raynaud’s phenomenon, puffy hands, arthralgia, and/or myopathy. However, fatal complications such as pulmonary arterial hypertension (PAH), interstitial lung disease (ILD), and/or renal crisis, which are associated with poor survival outcomes and increased mortality, can also be observed in patients with SSc [1].

In general, SSc is classified into two main subsets: diffuse cutaneous SSc (dcSSc) and limited cutaneous SSc (lcSSc). In lcSSc, skin thickness is limited to the face and areas distal to the elbows and knees, whereas extensive skin thickness is observed in dcSSc [2]. dcSSc is associated with a poorer prognosis compared to lcSSc. In the early onset of the disease, when skin thickness is restricted to the distal portions of the extremities, the classification of patients as having dcSSc or lcSSc is often difficult. In such cases, autoantibodies can help predict the future extent of skin thickness and patient prognosis [3,4].

The main autoantibodies in SSc that are included in the classification criteria for the diagnosis of SSc are anti-centromeres (ACA), anti-topoisomerase I (ATA) or anti-Scl-70, and anti-RNA polymerase III [5]. The amount of ATA is commonly reported to be between 18 and 85% [6,7,8,9,10,11] and is commonly present in 70–80% of Thai SSc patients [12,13]. The presence of ATA has been associated with dcSSc [7,14], pulmonary fibrosis or ILD [7,10,14], cardiac involvement [7], digital ulcers [14], hand deformities [6,12], a short duration of ILD in dcSSc [6], and a lower frequency of Raynaud’s phenomenon (RP) in lcSSc [6]. By comparison, the amount of ACA is reported to be between 7 and 40% [6,15,16], is associated with lcSSc [16] and PHT [7], and has a negative association with ILD and cardiac involvement [15,16]. In Thai patients with SSc, ACA was reported in less than 10% of cases and was negatively associated with hand deformity [12]. Although ATA was reported to be associated with the severe form of SSc (dcSSc) [7,14], neither ATA nor ACA was associated with the SSc subset among Thai patients and ATA was revealed to possibly be present in lcSSc [12,13].

Despite the association between ATA and extensive skin thickness, these antibodies are present in 15% of patients with lcSSc, in comparison with 55–91% of patients with dcSSc [11,12,13,17]. Patients with ATA-positive lcSSc may be distinguished as having another subset of the disease and might have a high risk of ILD, similar to ATA-positive dcSSc patients. Additionally, there might be an increased frequency of PHT in these lcSSc patients [18]. To date, no information exists on whether the clinical course of ATA-positive lcSSc is similar to that of ATA-positive dcSSc. Therefore, we investigated the clinical course of ATA-positive lcSSc in comparison with that of ATA-positive dcSSc among Thai patients, where dcSSc is the most common SSc subset. The findings from our study can be useful in guiding attending physicians to monitor and follow up on disease activity among patients with ATA-positive lcSSc.

## 2. Methods

A prospective cohort with a retrospective analysis was conducted among SSc patients who were diagnosed according to the 2013 ACR/EULAR classification criteria for SSc [5]; were over 18 years of age; and were followed up at the Scleroderma Clinic, Srinagarind Hospital, Khon Kaen University, Thailand, between 1 January 2014, and 31 December 2022. We included all patients who underwent autoantibody tests for ATA and ACA and were followed up at Srinagarind Hospital for at least 5 years in this study. We excluded patients with overlap syndromes and those who had ILD, PHT, renal crisis, and max-mRSS before entering the cohort.

Clinical data from the patients’ medical records and the SSc registry database were reviewed. The interval for the follow-up period depended on the disease severity, as determined by the attending physician at the Scleroderma Clinic. The clinical characteristics included the onset of first non-Raynaud’s SSc symptoms; the date of the last follow-up or the date of death; SSc subsets; clinical features of SSc (such as esophageal and gastrointestinal involvement, ILD, PHT, and renal crisis); the onset of these features after the onset of SSc; specific autoantibodies (ATA and ACA); patient status (death or survival); and causes of death, if applicable.

### 2.1. Operational Definitions

SSc was classified as the limited or diffuse type, per the classification in LeRoy et al. [2]. Onset of disease was considered the date of first non-Raynaud symptoms. A digital ulcer was defined as a painful denuded area with well-demarcated borders, located on the volar aspect of the fingers [19]. Hand deformity was defined when the finger joints have flexion contractures resembling claw deformities [20]. ILD was defined as interstitial fibrosis detected using high-resolution computed tomography (HRCT). PHT was diagnosed when the mean pulmonary arterial pressure (mPAP) was >20 mmHg at rest, with a pulmonary artery wedge pressure of ≤15 mmHg and a pulmonary vascular resistance of ≥3 wood units, as confirmed via right heart catheterization [21]. Severity of skin thickness was evaluated using the modified Rodnan skin score (mRSS) method, and the maximal mRSS was the highest mRSS recorded during follow-up. Esophageal involvement was defined as the presence of esophageal symptoms of SSc (i.e., esophageal dysphagia, heartburn, or reflux symptoms). Stomach involvement was defined as symptoms of early satiety or vomiting [22]. Intestinal involvement was determined by symptoms of diarrhea, bloating, malabsorption, constipation, ileus, or pseudo-intestinal obstruction. Cardiac involvement was defined as a left ventricular ejection fraction of ≤50% and/or pericardial effusion. Renal crisis was indicated when there was (a) a rapid, progressive rise in serum creatinine, (b) the abrupt onset of hypertension, and/or (c) microangiopathic hemolytic anemia. Weight loss was defined as unintentional loss of >5 percent of the usual body weight over 6–12 months [23].

The start date was the date of the first non-Raynaud’s symptom of SSc, and the end date was the conclusion of this study (31 December 2022). The duration of disease at the onset of ILD, PHT, renal crisis, and death was calculated by subtracting the date of first detection of ILD, PHT, renal crisis, and death from the date of the first non-Raynaud’s symptom of SSc. The status of patients who were lost to follow-up was retrieved from the government office, and the information was reviewed by a physician to ascertain the cause of death.

For autoantibody testing, the Euroline autoantibody test kit (Euroimmun AG, Lübeck, Germany), which includes SSc-specific antigens such as Scl-70 (from bovine and rabbit thymus) and centromere antigen subunits (CENP A and CENP B), was used. The sera of all participants and controls were tested using the Euroline immunoblot immunoglobulin G (IgG) technique. Initially, serum samples were diluted 1:100 and incubated with the test strips. In positive samples, specific IgG antibodies bound to the corresponding antigenic site. A second incubation was performed to detect the fixed antibodies using enzyme-labeled anti-human IgG, which displayed a color reaction. The reaction intensities were automatically graded using EurolineScan (Euroimmun AG, Lübeck, Germany), a computer software program that presents the result as either strongly positive, positive, borderline positive, or negative. Antibodies with borderline and weakly positive signal intensities were considered negative [6,12,24].

### 2.2. Statistical Analysis

Descriptive data are reported as proportions or percentages for categorical data and as median with interquartile range (IQR) or mean with standard deviation (SD) for continuous data. The clinical differences between lcSSc and dcSSc with ATA positivity were evaluated using the Chi-square test for categorical data, and Student’s t-test or the Wilcoxon rank-sum test for continuous data, as appropriate. The time to the development of events of interest (ILD, PHT, renal crisis, and maximal mRSS) was determined. The incidence of these events was assessed from the date of disease onset to the date of ILD, PHT, renal crisis, and maximal mRSS detection, with a 95% confidence interval (95% CI). The incidence of mortality with a 95% CI was assessed from the date of onset to the date of last follow-up or date of death. The Kaplan–Meier method was applied to estimate the probability of events of interest and mortality. A flexible parametric survival analysis using restricted mean survival time and the difference in restricted mean survival time (RMST) for ILD, PHT, renal crisis development, and maximal mRSS between the groups was assessed with a 95% CI and adjusted for age at onset, sex, mRSS, and organ involvement. Statistical significance was set at *p* < 0.05. All statistical analyses were performed using STATA version 16.0 (StataCorp, College Station, TX, USA).

## 3. Results

A total of 522 patients were included in this study, with a predominance of females over males, accounting for 319 cases compared to 203 cases (a ratio of 1.6:1). The mean age at the last visit and the mean disease duration were 59.7 ± 11.0 years and 7.5 ± 6.3 years, respectively. The most prevalent presentation in the Thai population was dcSSc with ATA positivity (dcSSc-posATA), at 61.5%, followed by lcSSc with ATA positivity (lcSSc-posATA), at 19.9%; lcSSc with ATA negativity (lcSSc-negATA), at 11.1%; and dcSSc with ATA negativity (dcSSc-negATA), at 7.5%. The proportion of females was lower in the dcSSc-posATA group than in the other groups (*p* = 0.004). The demographic data categorized by SSc subset and presence of ATA are presented in Table 1.

When comparing the clinical differences between lcSSc-negATA, lcSSc-posATA, dcSSc-posATA, and dcSSc-negATA, significant differences were found in the prevalence of digital gangrene (*p* = 0.04), telangiectasia (*p* = 0.01), salt and pepper skin (*p* < 0.001), tendon friction rub (*p* = 0.01), hand deformities (*p* < 0.001), mRSS (*p* < 0.001), and treatment with cyclophosphamide (*p* = 0.02) and mycophenolate mofetil (*p* = 0.03). However, no clinical differences were observed between the lcSSc-posATA and dcSSc-negATA groups, except for cyclophosphamide treatment (*p* = 0.01). The clinical differences between the groups are presented in Table 1.

When comparing the max-mRSS between lcSSc-negATA, lcSSc-posATA, dcSSc-posATA, and dcSSc-negATA, the maximum skin thickness assessed using mRSS at any time during follow-up was highest in the dcSSc-posATA group, with a mean mRSS of 19.7 ± 11.1 points, followed by dcSSc-negATA (12.2 ± 9.8 points), lcSSc-posATA (9.5 ± 7.4 points), and lcSSc-negATA (6.0 ± 5.9 points). A significant difference was observed between lcSSc-posATA and dcSSc-posATA (*p* < 0.001), but not for dcSSc-negATA (*p* = 0.95). The duration of disease at the maximum mRSS in lcSSc-posATA was comparable to that in dcSSc-posATA (1.8 years vs. 2.5 years; *p* = 0.17) but significantly shorter than that in the dcSSc-negATA group (1.8 vs. 6.4 years; *p* < 0.001). The number of patients with ILD did not differ between groups; however, the duration of disease at the onset of ILD was shorter among lcSSc-posATA patients than among dcSSc-negATA patients (*p* = 0.001) but comparable between lcSSc-posATA and dcSSc-posATA (*p* = 0.21). No differences were found in the number of patients with PHT or renal crisis, the duration of disease at the onset of PHT, and the duration of disease at the onset of renal crisis (Table 1).

### 3.1. Incidence of ILD

**Figure 1:**
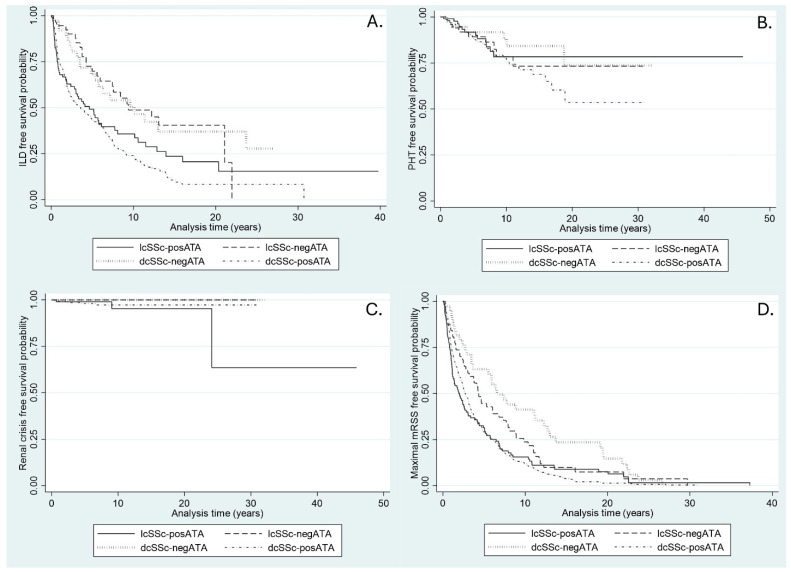
Kaplan–Meier graph of durations free from events in SSc patients categorized by SSc subset and the presence of ATA. (**A**) ILD-free. (**B**) PHT-free. (**C**) Renal crisis-free. (**D**) Maximal mRSS-free.

The incidence rate of ILD was highest in the dcSSc-posATA group, followed by lcSSc-posATA, lcSSc-negATA, and dcSSc-negATA. The median time to ILD development was shortest in the dcSSc-posATA group (Table 2). ILD-free survival in SSc patients, categorized by SSc subset and the presence of ATA, at 2, 5, 10, 15, and 20 years is presented as Kaplan–Meier graphs (Figure 1A). The ILD-free survival over time was significantly different between lcSSc-posATA and dcSSc-negATA (*p* = 0.03) but showed no difference when compared with dcSSc-posATA (*p* = 0.22).

After adjusting for sex, age at onset, PHT, renal crisis, and mRSS, the restricted mean free time to an ILD event was significantly longer in lcSSc-negATA at 5, 10, and 20 years than in lcSSc-posATA, with RMST differences of 1.2, 2.1, and 2.8 years, respectively (Table 3). The mean free time to ILD development was also significantly longer in patients with dcSSc-negATA than in the lcSSc-posATA group at 5 and 10 years, with RMST differences of 0.9 and 1.8 years, respectively. The restricted mean free time to ILD development and the differences in RMST between the groups at 5, 10, 20, and 40 years are presented in Table 3.

### 3.2. Incidence of PHT

The incidence rate of PHT was highest in the dcSSc-posATA group, followed by lcSSc-posATA, lcSSc-negATA, and dcSSc-negATA. The median time to PHT development was not determined because the follow-up time was too short to evaluate (Table 2). PHT-free survival in SSc patients, categorized by SSc subset and the presence of ATA, is presented as a Kaplan–Meier graph (Figure 1B). No significant differences were found in PHT-free survival over time between lcSSc-posATA and dcSSc-negATA (*p* = 0.68) or between lcSSc-posATA and dcSSc-posATA (*p* = 0.29). After adjusting for sex, age at onset, ILD, renal crisis, and mRSS, the restricted mean free time to PHT was comparable between the groups, and no difference was found in RMST at 5, 10, 20, and 40 years (Table 3).

### 3.3. Incidence of Renal Crisis

The incidence rate of renal crisis was low in all groups (Table 2). The median time to renal crisis development was not determined because the follow-up time was too short to evaluate. Renal crisis-free survival in SSc patients, categorized by SSc subset and the presence of ATA, is presented as a Kaplan–Meier graph (Figure 1B). No significant differences were found in renal crisis-free survival over time between lcSSc-posATA and dcSSc-negATA (*p* = 0.11), and between lcSSc-posATA and dcSSc-posATA (*p* = 0.73). After adjusting for sex, age at onset, ILD, PHT, and mRSS, the restricted mean free time to renal crisis at 20 years was 1.8 years longer in the lcSSc-negATA group and 1.8 years longer in the dcSSc-negATA group. At 40 years, it was 9.3 years longer in lcSSc-negATA and 9.3 years longer in dcSSc-negATA than in lcSSc-posATA (Table 3).

### 3.4. Time to Maximal mRSS

The median time to maximal mRSS was shortest in the lcSSc-posATA group (2.0 years), followed by dcSSc-posATA (2.7 years), lcSSc-negATA (4.3 years), and dcSSc-negATA (6.6 years) (Table 2). The maximal mRSS-free survival in SSc patients, categorized by SSc subset and the presence of ATA at 2, 5, 10, 15, and 20 years, is presented as Kaplan–Meier graphs (Figure 1A). The ILD-free survival over time was significantly different between lcSSc-posATA and dcSSc-negATA (*p* = 0.002) but showed no difference when compared with dcSSc-posATA (*p* = 0.77). After adjusting for sex, age at onset, presence of ILD, PHT, and renal crisis, the restricted mean free time to maximal mRSS in the lcSSc-posATA group was significantly shorter than that in the lcSSc-negATA group at 5 years, with a difference of 0.6 years. Patients with lcSSc-posATA also had a restricted mean free time to maximal mRSS shorter than dcSSc-negATA at 5, 10, 20, and 40 years, with differences of 1.2, 2.2, 2.9, and 3.1 years, respectively. However, the difference in RMST was comparable between the lcSSc-posATA and dcSSc-posATA groups at 5, 10, 20, and 40 years (Table 3).

### 3.5. Mortality Rate

**Figure 2:**
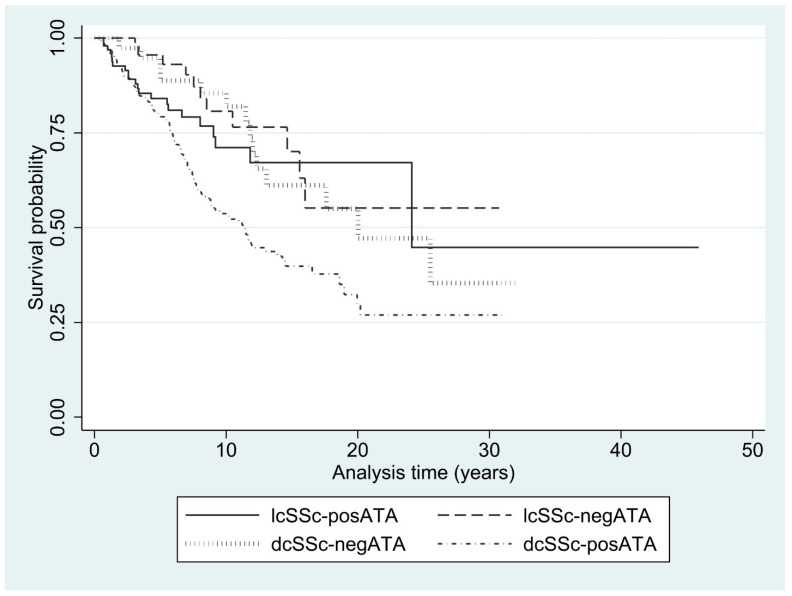
Kaplan–Meier graph of survival rate categorized by SSc subset and the presence of ATA.

Of the total 3,947.5 person-years, 171 patients died during follow-up, with an incidence rate of 4.3 per 100 person-years (95% CI 3.7–5.0). Most of the non-survivors were those with dcSSc-posATA (124 cases; 72.5%), followed by those with lcSSc-posATA (22 cases; 12.9%) and dcSSc-negATA (14 cases; 8.2%). The mortality rate was highest in patients with dcSSc-posATA, followed by lcSSc-posATA. A statistically significant difference was found in mortality rate over time between lcSSc-posATA and dcSSc-posATA (*p* = 0.01), but no difference was observed between lcSSc-posATA and dcSSc-negATA (*p* = 0.79). The mortality rates categorized by the SSc subset and presence of ATA are presented in Table 4. The Kaplan–Meier graph of survival rates categorized by the SSc subset and the presence of ATA is presented in Figure 2.

The causes of death were primarily non-SSc-related (92 cases; 53.8%), with pneumonia being the most common non-SSc-related cause of death (19 cases), followed by sepsis (11 cases). Nonspecific organ involvement was the most common cause of SSc-related death (28 cases), followed by cardiac involvement (24 cases). Renal crisis (three cases) was the most common cause of SSc-related death in the lcSSc-posATA group, and ILD (one case) was the most common cause of SSc-related death in the lcSSc-negATA group. Nonspecific organ involvement (24 cases) and cardiac involvement (5 cases) were the most common causes of SSc-related death in the dcSSc-posATA and dcSSc-negATA groups, respectively. The causes of death categorized by the SSc subset and presence of ATA are presented in Table 4.

## 4. Discussion

We conducted a large cohort study with a mean disease duration of approximately 8 years among the cases in the cohort. The cohort predominantly included individuals from the dcSSc subset, which is the most common SSc subset among Thai patients [13], whereas the lcSSc subset is common among Americans, Europeans, and some Asians [9,11,25,26,27,28,29,30,31,32]. We assessed the longitudinal data during follow-up using a survival analysis to define the clinical course of the disease in terms of the time to development of events of interest (ILD, PHT, renal crisis, maximal mRSS, and mortality). In addition, we used a flexible parametric survival analysis, including restricted mean survival time, to determine the difference in restricted mean survival time for the events of interest at 5, 10, 20, and 40 years of the disease between the lcSSc-pos ATA and other groups.

The proportion of patients with skin appendages such as salt and pepper skin; telangiectasia; calcinosis cutis; and tendon and muscular involvement, such as tendon friction rub and hand deformities, at the last follow-up in the lcSSc-posATA group was not different from that in the dcSSc-negATA group but was less frequent than that in the dcSSc-posATA group. These findings indicate the severity of both skin and musculoskeletal involvement in patients with extensive skin thickness who are positive for ATA.

Skin thickness progression was worse in dcSSc than in lcSSc, irrespective of the presence of ATA. The maximum skin thickness in patients with dcSSc was much higher than in patients with lcSSc. According to the definition of the SSc subset, dcSSc should have worse skin outcomes than lcSSc, which has less extensive thicknesses than the dcSSc subset. These results were expected. Therefore, it was not surprising that patients with dcSSc-pos ATA had the worst prognosis for skin outcomes compared with the other groups. However, the rate of skin progression, assessed by the time to reach the maximum mRSS after disease onset, was comparable between the lcSSc-pos ATA and dcSSc-pos ATA groups, with a median duration of 1.8 vs. 2.5 years. Additionally, the rate of skin progression in the lcSSc-pos ATA group was significantly shorter than in the dcSSc-neg ATA group (1.8 vs. 6.4 years). When we looked at the median survival of maximal mRSS, half of the patients with lcSSc-pos ATA and dcSSc-pos ATA had maximal mRSS in less than 3 years, whereas half of the patients with lcSSc and dcSSc negative for ATA had maximal mRSS at more than 4 years after onset. A previous study revealed that the higher the ATA level, the more rapid the skin thickness progression rate in the dcSSc subset [33]. Based on our observations, ATA might also be a potential marker for rapid skin thickness progression among the lcSSc subset, although the maximum skin thickness was not as high as in the dcSSc subset. We suggest that once ATA is positive in patients with early SSc, skin outcomes may not be favorable in the SSc subset, and treatment should not be delayed.

The clinical progression indicated a more rapid development of pulmonary-related disease in lcSSc-pos ATA, which exhibited faster clinical worsening compared to dcSSc-neg ATA. This finding was consistent with a previous study among Thai patients showing that patients, especially those in the dcSSc subset who were positive for ATA, had a significantly shorter median duration of disease until ILD detection compared to that of ATA-negative patients [6]. ATA is a well-known risk factor for ILD development and risk for ILD progression among SSc patients [34]. The pathogenesis of ILD is thought to begin with lung injury, which may be triggered by genetic susceptibility and/or infection. This process results in a loss of self-intolerance and an increase in antibody production against molecules of vascular origin. This leads to the activation of both innate and adaptive immune systems, which in turn activates fibroblasts and induces the production of pro-fibrotic cytokines, ultimately increasing the production of a collagen and extracellular matrix, causing lung fibrosis [35,36]. Antibodies that may play a major role in ILD include anti-endothelial cell antibodies [35], but no significant role for ATA has been proposed. Therefore, the pathogenic function of ATA in patients with ILD remains unclear. Based on the association between ATA and the rapid onset of ILD in SSc patients, regardless of the SSc subset, we suggest close monitoring for pulmonary complications, particularly in the early stages of the disease, for all ATA-positive SSc patients, even if they are asymptomatic for ILD. This approach aims to facilitate early treatment and subsequently prevent the extensive progression of lung fibrosis. However, we did not classify the severity of ILD, so we cannot provide information regarding the difference in ILD severity between the presence or absence of ATA among lcSSc and dcSSc patients.

Renal crisis is a common complication in ATA-positive patients, irrespective of the extent of skin thickness. Although our cohort had a low incidence of renal crisis, it was more frequently found in patients with ATA positivity in both the lcSSc and dcSSc subsets. Unfortunately, statistical limitations prevented us from testing for differences in incidence due to the low number of events. Renal crisis typically occurs within the first 1–4 years after disease onset, with a median time to renal crisis between 7.5 and 10 months [37,38]. However, the median time to renal crisis in our cohort was not available due to the low number of events during the follow-up period. Previous reports indicate that patients with dcSSc and rapidly progressive skin thickness are at risk for renal crisis [37,39,40]. Additionally, anti-RNA polymerase III antibodies and ATA have been reported as risk factors for renal crisis among patients with SSc [41,42]. Due to the low prevalence of anti-RNA polymerase III among Thai patients with SSc and its lack of clinical relevance [12], we did not include anti-RNA polymerase III in our analysis as a covariate. The higher incidence of renal crisis in the lcSSc-posATA group compared to the dcSSc-negATA group may be explained by the presence of ATA as a risk factor for renal crisis. However, whether any patients with renal crisis have both ATA and anti-RNA polymerase III positivity remains unclear. Our findings suggest that patients with lcSSc and ATA may be at risk of renal crisis, even if their skin thickness is not as extensive as that of patients with dcSSc.

Survival outcomes among lcSSc-posATA patients were worse than those among dcSSc-negATA patients but better than those among dcSSc-posATA patients during the first 10 years after disease onset. After this period, the survival of lcSSc-posATA patients improved and surpassed that of dcSSc-negATA patients. Renal crisis was a common cause of death among lcSSc-posATA patients, but not among dcSSc-negATA patients. Renal crisis commonly occurs within the first 4 years after disease onset [43,44] and is more frequently found in dcSSc than in lcSSc, with an estimated prevalence of 4–15% and 1–2%, respectively [42,43,45]. However, in our cohort, the incidence of renal crisis among patients with dcSSc was not as high as in previous studies (1.7% overall), and no renal crisis was observed among ATA-negative patients in either the dcSSc or lcSSc subsets.

Cardiac involvement was a common, organ-specific cause of death in both the dcSSc-posATA and dcSSc-negATA groups. The findings were similar to those in previous studies, which showed that cardiac involvement was the most common cause of SSc-related death, followed by ILD [13,46]. The causes of death related to SSc have changed over time; renal crisis has been the most common cause of death in the past 50 years [47], whereas ILD became the most common cause over the last 10–20 years [47,48]. Currently, cardiac involvement is the most common cause of death, according to our observations. This shift might be explained by the availability of therapeutic treatment options for renal crisis (angiotensin-converting enzyme inhibitors) [42] and ILD (immunosuppressants and anti-fibrotic agents) [49] but limited treatment options for cardiac involvement. However, we did not include cardiac involvement as an outcome of interest because the definition and screening tools for evaluating cardiac involvement in SSc—such as cardiac magnetic resonance imaging, electrocardiography, cardiac troponin-T levels, and N-terminal prohormone of brain natriuretic peptide levels—are facility-limited and not fully established. Additionally, the interval for evaluating cardiac involvement has not been described; abnormalities in these tests should be interpreted with caution, and other causes should be carefully ruled out. We suggest further studies focusing on symptomatic heart failure and cardiac arrhythmia as outcomes of interest in SSc.

Asymptomatic cardiac involvement has been reported in approximately 60% of cases in our previous cohort study, with diastolic dysfunction being the most common type of cardiac involvement. dcSSc was associated with asymptomatic cardiac involvement, but ATA did not show a significant association [50]. The incidence of symptomatic cardiac involvement after 2 years of follow-up among patients with asymptomatic cardiac involvement was 1.6 per 100 person-years, with PHT being the most common type of symptomatic cardiac involvement [51]. Myocardial inflammation is one type of cardiac involvement in SSc, particularly in patients with dcSSc. It is commonly found in early-onset SSc [52]. However, no biomarker is yet available for the detection of myocardial inflammation; cardiac magnetic resonance imaging remains the standard method. Once myocardial inflammation is detected, it may persist for up to 3 years of follow-up, and in some cases, it progresses to scarring [53]. The treatment options for myocardial inflammation are still limited. Although prednisolone at a dose of 30 mg/day has shown potential improvement in myocardial inflammation, it carries a risk of renal crisis [54]. A longitudinal follow-up of cardiac involvement with a median duration of 8.5 years showed an incidence of left ventricular systolic dysfunction of 0.88 per 100 person-years, which was most commonly found in the dcSSc subset [55]. Unfortunately, the study did not include ATA in its analysis. According to our literature review, a gap still exists in understanding the relationship between ATA and the long-term clinical course of cardiac involvement in SSc.

We analyzed the clinical course of disease by adjusting for variables such as sex, age at onset, mRSS, and internal organ involvement (including ILD, PHT, and renal crisis) in the model. However, we did not adjust for immunosuppressants due to variability in regimens and the intermittent nature of their prescription during follow-up. This may have led to a gap in our understanding on the potential therapeutic effects on the disease course. Additionally, immunosuppressants are mostly used in patients with high disease severity, particularly those with ILD or rapidly progressing extensive skin thickening. This limitation makes interpreting whether immunosuppressants can prevent or delay the onset of internal organ involvement challenging. Consequently, we cannot provide definitive information on the impact of immunosuppressants on the onset of internal organ involvement, as these factors were not included in our analysis. We suggest focusing future exploratory studies on the clinical course of the disease in SSc patients who receive early immunosuppressants and have not yet experienced internal organ involvement.

ATA is not the only specific autoantibody with clinical relevance to SSc; several others are also associated with distinct clinical characteristics. For example, the anti-centromere antibody (anti-CENP) is linked to the lcSSc subset or CREST syndrome [16] and PHT [7]. The anti-RNA polymerase III antibody has been associated with renal crisis, gastric antral vascular ectasia, and cancer [18]. The anti-Th/To antibody is reported to be related to PHT and ILD [7,16,56]. The anti-U3RNP antibody is associated with PHT [7,57,58], myositis [57,58], and gastrointestinal involvement [56], whereas the anti-U11/U12 RNP antibody is linked to ILD [59], PHT [18], and gastrointestinal involvement [59]. The anti-B23 antibody is associated with moderate to severe PHT [60], and both anti-Ku and anti-PM-Scl are related to scleroderma–polymyositis overlap syndrome [61]. However, these antibodies have low positivity rates and lacked significant clinical correlation among our Thai SSc patients. Therefore, they were not included as covariates in our statistical analysis.

Our study has some limitations: (a) It was a single-center cohort study, which may have limited the generalizability of our findings. Future validation in multi-center and/or multi-ethnic cohorts is recommended to enhance the external validity. (b) The ATA levels were not evaluated directly because the test became available at our center only in 2019. Therefore, as quantitative ATA data were not available during the study period, we relied on positive or negative ATA results in the analysis. Although the clinical association between ATA levels and SSc has been investigated among Thai patients [62], a larger sample size with long-term follow-up is needed for further evaluation. (c) Due to the low prevalence and limited clinical relevance of other specific autoantibody profiles (such as anti-RNA polymerase III, anti-Th/To, and anti-U3RNP) in Thai patients with SSc [12], these antibodies were not included as covariates in the statistical analysis, as noted above. (d) Our study included only ATA from the EUROLINE in the analysis and did not validate our results with other established methods such as immunoprecipitation or ELISA. Therefore, the findings may be limited to the use of the EUROLINE assay. Finally, (e) no data on the clinical course of cardiac involvement were available for comparison between groups, as discussed above.

The strengths of our study were as follows: (a) the inclusion of a large number of SSc patients, which provided a high level of analytical power; (b) the focus on major outcomes of interest, particularly the time to onset of organ involvement (such as ILD, PAH, renal crisis, and maximal mRSS), which may guide better care and monitoring of disease complications in patients with SSc; and (c) the use of a flexible parametric survival analysis to determine the restricted mean survival time for the events of interest over long-term follow-up periods (5, 10, 20, and 40 years), resulting in more practical implementation, appropriate interpretation, and reliable conclusions.

## 5. Conclusions

Patients with lcSSc-posATA had a similar risk of developing ILD to those with dcSSc, but they had a more rapid progression to pulmonary-related disease and faster clinical worsening compared to dcSSc-negATA. Skin thickness progression was worse in dcSSc than in lcSSc, regardless of the presence of ATA. The prognosis of lcSSc-posATA appeared to be better than that of dcSSc-posATA. Close monitoring of pulmonary complications, particularly in the early stages of the disease, for all ATA-positive SSc patients—regardless of the SSc subset—may facilitate early treatment and help prevent the extensive progression of pulmonary complications.

## 6. Key Messages

- ATA is a common antibody in the dcSSc subset but can also be found in lcSSc.
- Patients with lcSSc and positive for ATA had a similar ILD risk to those with dcSSc but experienced faster pulmonary worsening.
- ATA is positive in patients with early SSc, and skin outcomes may not be favorable in this SSc subset.

## Figures and Tables

**Table 1:** Demographic data categorized by SSc subset and the presence of ATA.

Data	Overall N = 522	lcSSc-negATA N = 58	lcSSc-posATA N = 104	dcSSc-negATA N = 39	dcSSc-posATA N = 321	*p*-Value Comparison Between dcSSc-negATA and lcSSc-posATA	*p*-Value Comparison Between dcSSc-posATA and lcSSc-posATA
Age at onset (years); mean ± SD	52.2 ± 12.2	51.2 ± 11.4	54.2 ± 12.4	47.0 ± 13.6	52.3 ± 11.9	0.003 *	0.16
Age at last visit (years); mean ± SD	59.7 ± 11.0	59.8 ± 10.7	61.5 ± 10.9	59.5 ± 12.0	59.1 ± 10.9	0.35	0.50
Female sex; N (%)	319 (61.1)	45 (77.6)	73 (70.2)	27 (69.2)	174 (54.2)	0.91	0.004 *
Disease duration (years); mean ± SD	7.5 ± 6.3	8.6 ± 6.7	7.2 ± 6.9	12.5 ± 8.2	6.8 ± 5.4	<0.001 *	0.99
BMI (kg/m^2^); mean ± SD	20.7 ± 3.8	21.3 ± 3.5	21.5 ± 3.7	19.3 ± 4.4	20.2 ± 3.8	0.95	0.14
SSc clinical features at the last follow-up							
Digital gangrene; N (%)	14 (2.7)	1 (1.72)	0 (0)	0 (0)	13 (4.1)	NA	0.04 *
Telangiectasia; N (%)	187 (35.8)	14 (24.1)	28 (26.9)	13 (33.3)	132 (41.1)	0.45	0.01 *
Calcinosis cutis; N (%)	40 (7.67)	4 (6.9)	3 (2.9)	2 (5.1)	31 (9.7)	0.52	0.03 *
Salt and pepper skin; N (%)	232 (44.4)	13 (22.4)	30 (28.9)	12 (30.8)	177 (55.1)	0.82	<0.001 *
Tendon friction rub; N (%)	71 (13.6)	2 (3.5)	7 (6.7)	4 (10.3)	58 (18.1)	0.48	0.01 *
Hand deformities; N (%)	199 (38.1)	6 (10.3)	22 (21.2)	9 (23.1)	162 (50.5)	0.80	<0.001 *
Muscle weakness; N (%)	26 (5.0)	2 (3.5)	6 (5.8)	1 (2.6)	17 (5.3)	0.43	0.85
Esophageal involvement; N (%)	220 (42.2)	19 (32.8)	40 (38.5)	16 (41.0)	145 (45.2)	0.78	0.23
Stomach involvement; N (%)	87 (16.7)	10 (17.2)	14 (13.5)	3 (7.7)	60 (18.7)	0.34	0.22
Intestine involvement; N (%)	89 (17.1)	10 (17.2)	15 (14.4)	9 (23.1)	55 (17.3)	0.22	0.52
mRSS (points); median (IQR)	3 (0–10)	0 (0–2)	2 (0–6)	2 (0–4)	6 (2–16)	0.83	<0.001 *
Immunosuppressants							
Cyclophosphamide; N (%)	61 (11.7)	2 (3.5)	7 (6.7)	0 (0)	52 (16.2)	0.01 *	0.02 *
Mycophenolate; N (%)	77 (14.8)	4 (6.9)	24 (23.1)	4 (10.3)	45 (14.0)	0.09	0.03 *
Methotrexate; N (%)	27 (5.2)	2 (3.5)	7 (6.7)	1 (2.6)	17 (5.3)	0.33	0.58
Interested events							
Maximum mRSS (points); mean ± SD	15.6 ± 11.2	6.0 ± 5.9	9.5 ± 7.4	12.2 ± 9.8	19.7 ± 11.1	0.95	<0.001 *
Duration of disease at maximum mRSS (years); median (IQR)	2.7 (1.0–6.7)	4.2 (1.5–8.9)	1.8 (0.8–5.5)	6.4 (2.4–13.0)	2.5 (0.9–5.7)	<0.001 *	0.17
ILD; N (%)	315 (60.3)	25 (43.1)	61 (58.6)	20 (51.3)	209 (65.1)	0.43	0.24
Duration of disease at onset of ILD (years); median (IQR)	1.9 (0.7–5.6)	4.2 (1.1–7.6)	1.0 (0.4–5.2)	4.8 (2.1–11.3)	1.8 (0.7–5.1)	0.001 *	0.21
PHT; N (%)	78 of 521 (15.0)	9 (15.5)	12 (11.5)	6 (15.4)	51 of 320 (15.9)	0.54	0.27
Duration of disease at onset of PHT (years); median (IQR)	4.08 (1.7–8.0)	4.3 (2.8–8.5)	3.0 (1.7–6.9)	4.9 (1.6–10.0)	4.2 (1.7–8.0)	0.08	0.30
Renal crisis; N (%)	9 of 522 (1.7)	0 (0)	3 (2.9)	0 (0)	8 (1.9)	0.28	0.62
Duration of disease at onset of renal crisis (years); median (IQR)	2.2 (1.0–9.0)	-	16.6 (9.0–24.1)	-	2.1 (1.0–2.2)	NA	0.05

* Statistical significance. lcSSc-negATA: limited cutaneous systemic sclerosis positive for the anti-topoisomerase I antibody; lcSSc-posATA: limited cutaneous systemic sclerosis negative for the anti-topoisomerase I antibody; dcSSc-negATA: diffuse cutaneous systemic sclerosis negative for the anti-topoisomerase I antibody; dcSSc-posATA: diffuse cutaneous systemic sclerosis positive for the anti-topoisomerase I antibody; mRSS: modified Rodnan skin score, 95%CI: 95% confidence interval, ILD: interstitial lung disease, PHT: pulmonary arterial hypertension.

**Table 2:** Incidence rate, median time to ILD development, and duration free from events categorized by SSc subset and the presence of ATA.

Data	Overall N = 522	lcSSc-negATA N = 58	lcSSc-posATA N = 104	dcSSc-negATA N = 39	dcSSc-posATA N = 321	*p*-Value Comparison Between dcSSc-negATA and lcSSc-posATA	*p*-Value Comparison Between dcSSc-posATA and lcSSc-posATA
ILD development							
Incidence rate of ILD (per 100 persons-year)	12.8 (11.5–14.4)	6.4 (4.3–9.6)	12.4 (9.6–16.0)	6.3 (4.1–9.7)	16.7 (14.5–19.1)		
Median survival time to ILD development (year)	4.9 (1.3–12.3)	9.3 (5.9–NA)	4.6 (1.0–2.8)	10.1 (4.9–23.7)	3.6 (2.4–4.9)		
ILD-free survival (%)						0.03 *	0.22
At 2 years	66.5	92.3	62.9	89.2	60.2		
At 5 years	49.6	69.8	49.2	68.3	43.9		
At 10 years	31.3	48.7	35.7	50.5	24.1		
At 15 years	19.3	40.5	23.6	37.0	10.8		
At 20 years	17.3	40.5	20.6	37.0	8.4		
PHT development							
Incidence rate of PHT (per 100 persons-year)	2.1 (1.7–2.7)	2.0 (1.0–3.8)	1.7 (0.9–3.0)	1.3 (0.5–3.0)	2.5 (1.9–3.2)		
Median survival time to PHT development (year)	NA	NA	NA	NA	NA		
PHT-free survival (%)						0.68	0.29
At 2 years	96.3	94.3	99.0	94.7	96.1		
At 5 years	89.8	89.2	91.3	91.7	88.9		
At 10 years	78.6	78.5	78.4	88.0	76.9		
At 15 years	73.2	73.3	78.4	84.2	68.8		
At 20 years	64.4	73.3	78.4	73.7	53.6		
Scleroderma renal crisis (SRC) development							
Incidence rate of SRC (per 100 persons-year)	0.2 (0.1–0.4)	NA	0.4 (0.01–0.1)	NA	0.02 (0.01–0.1)		
Median survival time to SRC development (year)	NA	NA	NA	NA	NA		
SRC-free survival (%)						0.11	0.73
At 2 years	99.4	100	98.9	100	99.3		
At 5 years	98.6	100	98.9	100	98.0		
At 10 years	97.6	100	95.3	100	97.3		
At 15 years	97.6	100	95.3	100	97.3		
At 20 years	97.6	100	95.3	100	97.3		
Maximum mRSS							
Median survival time to maximum mRSS development (year)	2.9 (1.1–6.8)	4.3 (2.8–6.8)	2.0 (1.2–2.8)	6.6 (3.5–12.3)	2.7 (2.4–3.1)		
Maximum mRSS-free survival (%)						0.002 *	0.77
At 2 years	60.4	71.8	49.2	81.6	59.4		
At 5 years	33.9	44.5	31.5	63.2	29.2		
At 10 years	16.3	23.7	15.6	41.2	12.2		
At 15 years	6.9	9.9	8.9	23.5	3.8		
At 20 years	4.1	7.4	7.6	14.7	1.4		

* Statistical significance. lcSSc-negATA, limited cutaneous systemic sclerosis positive for the anti-topoisomerase I antibody; lcSSc-posATA, limited cutaneous systemic sclerosis negative for the anti-topoisomerase I antibody; dcSSc-negATA, diffuse cutaneous systemic sclerosis negative for the anti-topoisomerase I antibody; dcSSc-posATA, diffuse cutaneous systemic sclerosis positive for the anti-topoisomerase I antibody; 95%CI, 95% confidence interval; ILD, interstitial lung disease; PHT, pulmonary arterial hypertension; SRC, scleroderma renal crisis; mRSS, modified Rodnan skin score.

**Table 3:** Differences in Restricted mean free time to ILD development between groups adjusted by age at onset, sex, PHT diagnosis, renal crisis, and maximum mRSS.

Landmark (Years)	RMST (95%CI)				RMST Difference Between Groups 1 and 2	RMST Difference Between Groups 3 and 2	RMST Difference Between Groups 4 and 2
	Group 1 lcSSc-negATA	Group 2 lcSSc-posATA	Group 3 dcSSc-negATA	Group 4 dcSSc-posATA			
ILD							
5 years	4.1 (3.7 to 4.5)	2.9 (2.5 to 3.3)	3.8 (3.3 to 4.4)	3.2 (3.0 to 3.4)	1.2 (0.6 to 1.8) *	0.9 (0.3 to 1.6) *	0.3 (−1.1 to 0.7)
10 years	6.7 (5.7 to 7.7)	4.5 (3.8 to 5.3)	6.3 (5.1 to 7.5)	4.8 (4.4 to 5.2)	2.1 (0.9 to 3.4) *	1.8 (0.4 to 3.2) *	0.3 (−0.6 to 1.2)
20 years	9.2 (7.1 to 11.2)	6.3 (4.9 to 7.7)	8.9 (6.6 to 11.3)	6.3 (5.5 to 7.0)	2.8 (0.4 to 5.2) *	2.6 (−0.1 to 5.3)	−0.1 (−1.7 to 1.6)
40 years	10.4 (7.3 to 13.5)	7.7 (5.4 to 10.1)	10.8 (7.0 to 14.0)	7.0 (5.8 to 8.2)	2.6 (−1.3 to 6.5)	3.0 (−1.5 to 7.6)	−0.8 (−3.4 to 1.9)
PHT							
5 years	4.6 (4.3 to 5.0)	4.8 (4.6 to 4.9)	4.7 (4.4 to 5.1)	4.7 (4.6 to 4.8)	−0.1 (−0.5 to 0.2)	−0.1 (−0.5 to 0.3)	−0.5 (−0.2 to 0.1)
10 years	8.6 (7.7 to 9.5)	9.1 (8.6 to 9.6)	8.8 (7.8 to 9.8)	8.7 (8.4 to 9.1)	−0.4 (−1.5 to 0.6)	−0.2 (−1.3 to 0.8)	−0.3 (−1.0 to 0.3)
20 years	15.4 (12.9 to 17.9)	16.6 (14.8 to 18.4)	15.9 (13.2 to 18.6)	14.9 (13.7 to 16.1)	−1.1 (−4.1 to 1.9)	−0.7 (−3.8 to 2.5)	−1.7 (−3.8 to 0.5)
40 years	26.0 (18.2 to 33.7)	28.3 (21.5 to 35.2)	26.8 (18.5 to 35.2)	22.5 (18.0 to 21.0)	−2.4 (−11.7 to 7.0)	−1.5 (−11.6 to 8.6)	−5.8 (−13.1 to 1.5)
Renal crisis							
5 years	5.0 (5.0 to 5.0)	4.9 (4.8 to 5.0)	5.0 (5.0 to 5.0)	4.9 (4.9 to 5.0)	0.1 (−0.04 to −0.2)	0.1 (−0.04 to −0.2)	0.02 (−0.1 to 0.2)
10 years	10.0 (10.0 to 10.0)	9.7 (9.3 to 10.1)	10.0 (10.0 to 10.0)	9.8 (9.7 to 10.0)	0.3 (−0.1 to 0.7)	0.3 (−0.1 to 0.7)	0.2 (−0.3 to 0.6)
20 years	20.0 (20.0 to 20.0)	18.2 (16.3 to 20.0)	20.0 (20.0 to 20.0)	19.1 (18.3 to 20.0)	1.8 (0.1 to 3.6) *	1.8 (0.1 to 3.6) *	1.0 (−1.0 to 2.9)
40 years	40.0 (40.0 to 40.0)	30.7 (21.7 to 39.7)	40.0 (40.0 to 40.0)	35.6 (28.9 to 42.3)	9.3 (0.4 to 18.2) *	9.3 (0.4 to 18.2) *	4.9 (−4.3 to 14.1)
Maximum mRSS							
5 years	3.3 (2.8 to 3.7)	2.7 (2.3 to 3.0)	3.8 (3.3 to 4.4)	2.8 (2.7 to 3.0)	0.6 (0.1 to 1.2) *	1.2 (0.6 to 1.8) *	0.2 (−0.2 to 0.6)
10 years	4.8 (3.9 to 5.6)	3.8 (3.2 to 4.4)	6.0 (4.8 to 7.1)	3.8 (3.4 to 4.1)	1.0 (−0.1 to 2.0)	2.2 (0.9 to 3.4) *	−0.03 (−0.7 to 0.7)
20 years	5.8 (4.5 to 7.1)	4.8 (3.8 to 5.8)	7.7 (5.9 to 9.5)	4.2 (3.8 to 4.7)	1.1 (−0.6 to 2.7)	2.9 (0.8 to 5.0) *	−0.5 (−1.6 to 0.6)
40 years	6.2 (4.6 to 7.7)	5.2 (3.9 to 6.6)	8.3 (6.1 to 10.5)	4.3 (3.8 to 4.8)	0.9 (−1.1 to 3.0)	3.1 (0.5 to 5.7) *	−0.9 (−2.3 to 0.5)

* Statistical significance. RMST, restrict mean survival time; ILD, interstitial lung disease; PHT, pulmonary arterial hypertension; mRSS, modified Rodnan skin score.

**Table 4:** Mortality rate and causes of death categorized by SSc subset and the presence of ATA.

Data	Overall N = 171	lcSSc-negATA N = 11	lcSSc-posATA N = 22	dcSSc-negATA N = 14	dcSSc-posATA N = 124
**Mortality rate per 100-person-years (95%CI)**	4.3 (3.7-5.0)	2.2 (1.2-3.9)	2.9 (1.9-4.4)	2.8 (1.7-4.8)	5.7 (4.7-6.7)
2-year survival (%)	93.5	100	92.7	100	91.9
5-year survival (%)	83.0	95.6	84.2	91.6	79.0
10-year survival (%)	63.0	80.7	71.3	85.4	53.4
15-year survival (%)	49.6	70.1	67.3	61.1	39.6
20-year survival (%)	41.3	55.2	67.3	55.0	29.5
**Causes of death**					
SSc related death; N (%)	79 (46.2)	1 (9.1)	9 (40.1)	9 (64.3)	60 (48.4)
Nonspecific organ; N (%)	28 (16.4)	0 (0.0)	1 (4.6)	3 (21.3)	24 (19.4)
ILD; N (%)	11 (6.4)	1 (9.1)	1 (4.6)	1 (7.2)	8 (6.5)
Cardiac involvement; N (%)	24 (14.0)	0 (0.0)	2 (9.1)	5 (35.7)	17 (13.7)
PHT; N (%)	7 (4.1)	0 (0.0)	1 (4.6)	0 (0.0)	6 (4.8)
Renal crisis; N (%)	8 (4.7)	0 (0.0)	3 (16.4)	0 (0.0)	5 (4.0)
Gastrointestinal; N (%)	1 (0.6)	0 (0.0)	1 (4.6)	0 (0.0)	0 (0.0)
SSc non related death	92 (53.8)	10 (90.9)	13 (59.1)	5 (35.7)	64 (51.6)
Cancer; N (%)	2 (0.0)	0 (0.0)	0 (0.0)	0 (0.0)	2 (1.6)
Pneumonia; N (%)	19 (11.1)	0 (0.0)	2 (8.7)	1 (7.1)	16 (12.9)
Sepsis; N (%)	11 (6.4)	2 (18.2)	1 (4.4)	1 (7.1)	6 (5.7)
CAD; N (%)	6 (3.5)	1 (9.1)	0 (0.0)	0 (0.0)	5 (4.0)
Natural death; N (%)	1 (0.6)	0 (0.0)	0 (0.0)	0 (0.0)	1 (0.8)
CKD; N (%)	2 (1.2)	0 (0.0)	1 (4.4)	0 (0.0)	1 (0.8)
Liver disease; N (%)	3 (1.7)	1 (9.1)	0 (0.0)	0 (0.0)	2 (1.6)
ICH; N (%)	2 (1.2)	0 (0.0)	0 (0.0)	0 (0.0)	2 (1.6)
other cause; N (%)	46 (26.9)	6 (54.5)	9 (39.1)	3 (21.4)	28 (22.6)

95%CI: 95% confidence interval, lcSSc-negATA: limited cutaneous systemic sclerosis with anti-topoisomerase I antibody-positive, lcSSc-posATA: limited cutaneous systemic sclerosis with anti-topoisomerase I antibody-negative. dcSSc-negATA: diffuse cutaneous systemic sclerosis with anti-topoisomerase I antibody negative; dcSSc-posATA: diffuse cutaneous systemic sclerosis with anti-topoisomerase I antibody positive; ILD, interstitial lung disease; PHT, pulmonary arterial hypertension; CAD, coronary artery disease; CKD, chronic kidney disease; ICH, intracranial hemorrhage.

## Data Availability

The datasets used and/or analyzed during the current study are available from the corresponding author upon reasonable request.

## References

[1] Al-Dhaher F.F., Pope J.E., Ouimet J.M. (2010). Determinants of Morbidity and Mortality of Systemic Sclerosis in Canada. Semin. Arthritis Rheum..

[2] LeRoy E.C., Black C., Fleischmajer R., Jablonska S., Krieg T., Medsger T.A., Rowell N., Wollheim F. (1988). Scleroderma (Systemic Sclerosis): Classification, Subsets and Pathogenesis. J. Rheumatol..

[3] Kuwana M., Kaburaki J., Okano Y., Tojo T., Homma M. (1994). Clinical and Prognostic Associations Based on Serum Antinuclear Antibodies in Japanese Patients with Systemic Sclerosis. Arthritis Rheum..

[4] Kuwana M. (2017). Circulating Anti-Nuclear Antibodies in Systemic Sclerosis: Utility in Diagnosis and Disease Subsetting. J. Nippon. Med. Sch..

[5] van den Hoogen F., Khanna D., Fransen J., Johnson S.R., Baron M., Tyndall A., Matucci-Cerinic M., Naden R.P., Medsger T.A., Carreira P.E. (2013). 2013 Classification Criteria for Systemic Sclerosis: An American College of Rheumatology/European League against Rheumatism Collaborative Initiative. Arthritis Rheum..

[6] Foocharoen C., Suwannachat P., Netwijitpan S., Mahakkanukrauh A., Suwannaroj S., Nanagara R. (2016). Scleroderma Research Group Clinical Differences between Thai Systemic Sclerosis Patients with Positive versus Negative Anti-Topoisomerase I. Int. J. Rheum. Dis..

[7] Steen V.D. (2005). Autoantibodies in Systemic Sclerosis. Semin. Arthritis Rheum..

[8] Graf S.W., Hakendorf P., Lester S., Patterson K., Walker J.G., Smith M.D., Ahern M.J., Roberts-Thomson P.J. (2012). South Australian Scleroderma Register: Autoantibodies as Predictive Biomarkers of Phenotype and Outcome. Int. J. Rheum. Dis..

[9] Hashimoto A., Endo H., Kondo H., Hirohata S. (2012). Clinical Features of 405 Japanese Patients with Systemic Sclerosis. Mod. Rheumatol..

[10] Wang J., Assassi S., Guo G., Tu W., Wu W., Yang L., Xiao R., Zhao Y., Chu H., Liu J. (2013). Clinical and Serological Features of Systemic Sclerosis in a Chinese Cohort. Clin. Rheumatol..

[11] Poormoghim H., Moghadam A.S., Moradi-Lakeh M., Jafarzadeh M., Asadifar B., Ghelman M., Andalib E. (2013). Systemic Sclerosis: Demographic, Clinical and Serological Features in 100 Iranian Patients. Rheumatol. Int..

[12] Foocharoen C., Watcharenwong P., Netwijitpan S., Mahakkanukrauh A., Suwannaroj S., Nanagara R. (2017). Relevance of Clinical and Autoantibody Profiles in Systemic Sclerosis among Thais. Int. J. Rheum. Dis..

[13] Foocharoen C., Peansukwech U., Mahakkanukrauh A., Suwannaroj S., Pongkulkiat P., Khamphiw P., Nanagara R. (2020). Clinical Characteristics and Outcomes of 566 Thais with Systemic Sclerosis: A Cohort Study. Int. J. Rheum. Dis..

[14] Villalta D., Imbastaro T., Di Giovanni S., Lauriti C., Gabini M., Turi M.C., Bizzaro N. (2012). Diagnostic Accuracy and Predictive Value of Extended Autoantibody Profile in Systemic Sclerosis. Autoimmun. Rev..

[15] Kayser C., Fritzler M.J. (2015). Autoantibodies in Systemic Sclerosis: Unanswered Questions. Front. Immunol..

[16] Moinzadeh P., Nihtyanova S.I., Howell K., Ong V.H., Denton C.P. (2012). Impact of Hallmark Autoantibody Reactivity on Early Diagnosis in Scleroderma. Clin. Rev. Allergy Immunol..

[17] Srivastava N., Hudson M., Tatibouet S., Wang M., Baron M., Fritzler M.J. (2015). Canadian Scleroderma Research Group (CSRG) Thinking Outside the Box-The Associations with Cutaneous Involvement and Autoantibody Status in Systemic Sclerosis Are Not Always What We Expect. Semin. Arthritis Rheum..

[18] Stochmal A., Czuwara J., Trojanowska M., Rudnicka L. (2020). Antinuclear Antibodies in Systemic Sclerosis: An Update. Clin. Rev. Allergy Immunol..

[19] Korn J.H., Mayes M., Matucci Cerinic M., Rainisio M., Pope J., Hachulla E., Rich E., Carpentier P., Molitor J., Seibold J.R. (2004). Digital Ulcers in Systemic Sclerosis: Prevention by Treatment with Bosentan, an Oral Endothelin Receptor Antagonist. Arthritis Rheum..

[20] Young A., Namas R., Dodge C., Khanna D. (2016). Hand Impairment in Systemic Sclerosis: Various Manifestations and Currently Available Treatment. Curr. Treatm Opt. Rheumatol..

[21] Simonneau G., Montani D., Celermajer D.S., Denton C.P., Gatzoulis M.A., Krowka M., Williams P.G., Souza R. (2019). Haemodynamic Definitions and Updated Clinical Classification of Pulmonary Hypertension. Eur. Respir. J..

[22] Savarino E., Furnari M., de Bortoli N., Martinucci I., Bodini G., Ghio M., Savarino V. (2014). Gastrointestinal Involvement in Systemic Sclerosis. Presse Med..

[23] Wong C.J. (2014). Involuntary Weight Loss. Med. Clin. North. Am..

[24] Sujau I., Ng C.T., Sthaneshwar P., Sockalingam S., Cheah T.E., Yahya F., Jasmin R. (2015). Clinical and Autoantibody Profile in Systemic Sclerosis: Baseline Characteristics from a West Malaysian Cohort. Int. J. Rheum. Dis..

[25] Roberts-Thomson P.J., Jones M., Hakendorf P., Kencana Dharmapatni A.A., Walker J.G., MacFarlane J.G., Smith M.D., Ahern M.J. (2001). Scleroderma in South Australia: Epidemiological Observations of Possible Pathogenic Significance. Intern. Med. J..

[26] Englert H., Small-McMahon J., Davis K., O’Connor H., Chambers P., Brooks P. (1999). Systemic Sclerosis Prevalence and Mortality in Sydney 1974-88. Aust. N. Z. J. Med..

[27] Tamaki T., Mori S., Takehara K. (1991). Epidemiological Study of Patients with Systemic Sclerosis in Tokyo. Arch. Dermatol. Res..

[28] Geirsson A.J., Steinsson K., Guthmundsson S., Sigurthsson V. (1994). Systemic Sclerosis in Iceland. A Nationwide Epidemiological Study. Ann. Rheum. Dis..

[29] Ferri C., Valentini G., Cozzi F., Sebastiani M., Michelassi C., La Montagna G., Bullo A., Cazzato M., Tirri E., Storino F. (2002). Systemic Sclerosis: Demographic, Clinical, and Serologic Features and Survival in 1,012 Italian Patients. Medicine.

[30] Tyndall A., Mueller-Ladner U., Matucci-Cerinic M. (2005). Systemic Sclerosis in Europe: First Report from the EULAR Scleroderma Trials And Research (EUSTAR) Group Database. Ann. Rheum. Dis..

[31] Delisle V.C., Hudson M., Baron M., Thombs B.D., The Canadian Scleroderma Research Group A (2014). Sex and Time to Diagnosis in Systemic Sclerosis: An Updated Analysis of 1,129 Patients from the Canadian Scleroderma Research Group Registry. Clin. Exp. Rheumatol..

[32] Reveille J.D., Fischbach M., McNearney T., Friedman A.W., Aguilar M.B., Lisse J., Fritzler M.J., Ahn C., Arnett F.C. (2001). GENISOS Study Group Systemic Sclerosis in 3 US Ethnic Groups: A Comparison of Clinical, Sociodemographic, Serologic, and Immunogenetic Determinants. Semin. Arthritis Rheum..

[33] Perera A., Fertig N., Lucas M., Rodriguez-Reyna T.S., Hu P., Steen V.D., Medsger T.A. (2007). Clinical Subsets, Skin Thickness Progression Rate, and Serum Antibody Levels in Systemic Sclerosis Patients with Anti-Topoisomerase I Antibody. Arthritis Rheum..

[34] Martín-López M., Carreira P.E. (2023). The Impact of Progressive Pulmonary Fibrosis in Systemic Sclerosis-Associated Interstitial Lung Disease. J. Clin. Med..

[35] Liakouli V., Ciancio A., Del Galdo F., Giacomelli R., Ciccia F. (2024). Systemic Sclerosis Interstitial Lung Disease: Unmet Needs and Potential Solutions. Nat. Rev. Rheumatol..

[36] Nihtyanova S.I., Denton C.P. (2020). Pathogenesis of Systemic Sclerosis Associated Interstitial Lung Disease. J. Scleroderma Relat. Disord..

[37] Penn H., Howie A.J., Kingdon E.J., Bunn C.C., Stratton R.J., Black C.M., Burns A., Denton C.P. (2007). Scleroderma Renal Crisis: Patient Characteristics and Long-Term Outcomes. QJM.

[38] Foocharoen C., Mahakkanukrauh A., Suwannaroj S., Nanagara R. (2016). Prevalence and Clinical Features of Acute Kidney Injury in Thai Systemic Sclerosis Patients. KKU Med. J..

[39] Teixeira L., Mouthon L., Mahr A., Berezné A., Agard C., Mehrenberger M., Noël L.-H., Trolliet P., Frances C., Cabane J. (2008). Mortality and Risk Factors of Scleroderma Renal Crisis: A French Retrospective Study of 50 Patients. Ann. Rheum. Dis..

[40] Domsic R.T., Rodriguez-Reyna T., Lucas M., Fertig N., Medsger T.A. (2011). Skin Thickness Progression Rate: A Predictor of Mortality and Early Internal Organ Involvement in Diffuse Scleroderma. Ann. Rheum. Dis..

[41] Nguyen B., Assassi S., Arnett F.C., Mayes M.D. (2010). Association of RNA Polymerase III Antibodies with Scleroderma Renal Crisis. J. Rheumatol..

[42] Foocharoen C., Tonsawan P., Pongkulkiat P., Anutrakulchai S., Mahakkanukrauh A., Suwannaroj S. (2023). Management Review of Scleroderma Renal Crisis: An Update with Practical Pointers. Mod. Rheumatol..

[43] Steen V.D. (2003). Scleroderma Renal Crisis. Rheum. Dis. Clin. North. Am..

[44] Dolnikov K., Milo G., Assady S., Dragu R., Braun-Moscovici Y., Balbir-Gurman A. (2020). Scleroderma Renal Crisis as an Early Presentation of Systemic Sclerosis. Isr. Med. Assoc. J..

[45] Muangchan C., Baron M., Pope J., Canadian Scleroderma Research Group (2013). The 15% Rule in Scleroderma: The Frequency of Severe Organ Complications in Systemic Sclerosis. A Systematic Review. J. Rheumatol..

[46] Elhai M., Meune C., Boubaya M., Avouac J., Hachulla E., Balbir-Gurman A., Riemekasten G., Airò P., Joven B., Vettori S. (2017). Mapping and Predicting Mortality from Systemic Sclerosis. Ann. Rheum. Dis..

[47] Steen V.D., Medsger T.A. (2007). Changes in Causes of Death in Systemic Sclerosis, 1972-2002. Ann. Rheum. Dis..

[48] Rubio-Rivas M., Royo C., Simeón C.P., Corbella X., Fonollosa V. (2014). Mortality and Survival in Systemic Sclerosis: Systematic Review and Meta-Analysis. Semin. Arthritis Rheum..

[49] Raghu G., Montesi S.B., Silver R.M., Hossain T., Macrea M., Herman D., Barnes H., Adegunsoye A., Azuma A., Chung L. (2024). Treatment of Systemic Sclerosis-Associated Interstitial Lung Disease: Evidence-Based Recommendations. An Official American Thoracic Society Clinical Practice Guideline. Am. J. Respir. Crit. Care Med..

[50] Foocharoen C., Pussadhamma B., Mahakkanukrauh A., Suwannaroj S., Nanagara R. (2015). Asymptomatic Cardiac Involvement in Thai Systemic Sclerosis: Prevalence and Clinical Correlations with Non-Cardiac Manifestations (Preliminary Report). Rheumatology.

[51] Pussadhamma B., Mahakkanukrauh A., Suwannaroj S., Nanagara R., Foocharoen C. (2021). Clinical Outcomes of Asymptomatic Cardiac Involvement in Systemic Sclerosis Patients After a 2-Year Follow-Up (Extended Study). Am. J. Med. Sci..

[52] Tipparot T., Foocharoen C., Mahakkanukrauh A., Suwannaroj S., Nanagara R., Pussadhamma B., Chaosuwannakit N. (2019). Clinical and Laboratory Predictions of Myocardial Inflammation as Detected by Cardiac Magnetic Resonance Imaging in Patients with Systemic Sclerosis: A Pilot Study. Int. J. Rheum. Dis..

[53] Mahakkanukrauh A., Foocharoen C., Chaosuwannakit N., Suwannaroj S., Pongkulkiat P., Onchan T., Pussadhamma B. (2024). Outcomes of Myocarditis in Systemic Sclerosis: A 3-Year Follow-Up. Rheumatol. Immunol. Res..

[54] Pussadhamma B., Tipparot T., Chaosuwannakit N., Mahakkanukrauh A., Suwannaroj S., Nanagara R., Foocharoen C. (2020). Clinical Outcomes of Myocarditis after Moderate-Dose Steroid Therapy in Systemic Sclerosis: A Pilot Study. Int. J. Rheumatol..

[55] Werakiat J., Pussadhamma B., Mahakkanukrauh A., Suwannaroj S., Foocharoen C. (2024). Clinical Courses and Predictors of Left Ventricular Systolic Dysfunction in Systemic Sclerosis: A Cohort Study. Rheumatol. Immunol. Res..

[56] Mehra S., Walker J., Patterson K., Fritzler M.J. (2013). Autoantibodies in Systemic Sclerosis. Autoimmun. Rev..

[57] Aggarwal R., Lucas M., Fertig N., Oddis C.V., Medsger T.A. (2009). Anti-U3 RNP Autoantibodies in Systemic Sclerosis. Arthritis Rheum..

[58] Tormey V.J., Bunn C.C., Denton C.P., Black C.M. (2001). Anti-Fibrillarin Antibodies in Systemic Sclerosis. Rheumatology.

[59] Fertig N., Domsic R.T., Rodriguez-Reyna T., Kuwana M., Lucas M., Medsger T.A., Feghali-Bostwick C.A. (2009). Anti-U11/U12 RNP Antibodies in Systemic Sclerosis: A New Serologic Marker Associated with Pulmonary Fibrosis. Arthritis Rheum..

[60] Ulanet D.B., Wigley F.M., Gelber A.C., Rosen A. (2003). Autoantibodies against B23, a Nucleolar Phosphoprotein, Occur in Scleroderma and Are Associated with Pulmonary Hypertension. Arthritis Rheum..

[61] Rozman B., Cucnik S., Sodin-Semrl S., Czirják L., Varjú C., Distler O., Huscher D., Aringer M., Steiner G., Matucci-Cerinic M. (2008). Prevalence and Clinical Associations of Anti-Ku Antibodies in Patients with Systemic Sclerosis: A European EUSTAR-Initiated Multi-Centre Case-Control Study. Ann. Rheum. Dis..

[62] Mulalin K., Mahakkanukrauh A., Suwannaroj S., Pongkulkiat P., Onchan T., Kasa S., Foocharoen C. (2024). Levels of Anti-Topoisomerase I Antibody Correlated with Short Onset of Cardiopulmonary Involvement in Thai Systemic Sclerosis Patients. Sci. Rep..

